# Normobaric hyperoxia alleviates complement C3‐mediated synaptic pruning and brain injury after intracerebral hemorrhage

**DOI:** 10.1111/cns.14694

**Published:** 2024-03-26

**Authors:** Moxin Wu, Kai Chen, Yasong Zhao, Min Jiang, Bing Bao, Wenmin Yu, Zhiying Chen, Xiaoping Yin

**Affiliations:** ^1^ Department of Medical Laboratory Affiliated Hospital of Jiujiang University Jiujiang Jiangxi China; ^2^ Jiujiang Clinical Precision Medicine Research Center Jiujiang Jiangxi China; ^3^ Department of Dermatology, Traditional Chinese and Western Medicine Hospital of Wuhan, Tongji Medical College Huazhong University of Science and Technology Wuhan China; ^4^ Department of Neurology Affiliated Hospital of Jiujiang University Jiujiang China; ^5^ Jiangxi Provincial Key Laboratory of Cell Precision Therapy, School of Basic Medical Sciences Jiujiang University Jiujiang Jiangxi China

**Keywords:** complement component 3, intracerebral hemorrhage, microglia, normobaric hyperoxia, synaptic pruning

## Abstract

**Background:**

Intracerebral hemorrhage (ICH) is a common cerebrovascular disease, and the complement cascade exacerbates brain injury after ICH. As the most abundant component of the complement system, complement component 3 (C3) plays essential roles in all three complement pathways. However, the effects of C3 on neurological impairment and brain injury in ICH patients and the related mechanism have not been fully elucidated. Normobaric hyperoxia (NBO) is regarded as a treatment for ICH patients, and recent clinical studies also have confirmed the neuroprotective role of NBO against acute ICH‐mediated brain damage, but the underlying mechanism still remains elusive.

**Aims:**

In the present study, we investigated the effects of complement C3 on NBO‐treated ICH patients and model mice, and the underlying mechanism of NBO therapy in ICH‐mediated brain injury.

**Results:**

Hemorrhagic injury resulted in the high plasma C3 levels in ICH patients, and the plasma C3 levels were closely related to hemorrhagic severity and clinical outcomes after ICH. BO treatment alleviated neurologic impairments and rescued the hemorrhagic‐induced increase in plasma C3 levels in ICH patients and model mice. Moreover, the results indicated that NBO exerted its protective effects of on brain injury after ICH by downregulating the expression of C3 in microglia and alleviating microglia‐mediated synaptic pruning.

**Conclusions:**

Our results revealed that NBO exerts its neuroprotective effects by reducing C3‐mediated synaptic pruning, which suggested that NBO therapy could be used for the clinical treatment of ICH.

## Graphical abstract

The plasma C3 levels were closely related to the hemorrhagic severity and the clinical outcomes after ICH. The protective effects of NBO treatment on brain injury after ICH were exerted via downregulating the expression of C3 in microglia and alleviating microglia‐mediated synaptic pruning.

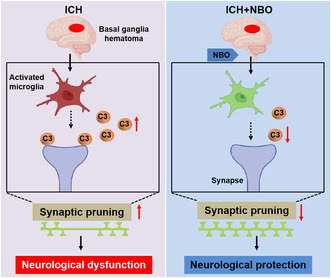

## 1 INTRODUCTION

Intracerebral hemorrhage (ICH) is the second most common stroke type and accounts for approximately 15% of all strokes; it is characterized by blood vessel rupture within the brain parenchyma and has a high mortality rate.^1^, ^2^ After ICH onset, the release of cytotoxic blood components following the destruction of brain structures, results in neuronal cell death and synaptic dysfunction, with high disability rates, and functional independence at 6 months post onset is observed in only 20% of patients.^3^, ^4^ ICH is an enormous economic and mental burden on patients and their families, and is becoming a serious global health problem.^5^ Therefore, novel treatment strategies for clinical rehabilitation therapy and alleviation of cognitive impairment in ICH patients are needed.

The complement system is an essential part of the innate immune system and has typical functions in host defense and immunomodulation.^6^ The complement cascade can be activated via three pathways (the classical, lectin, or alternative pathway) and contributes to inflammation, neuronal cell death and brain injury after ICH.^7^ As the convergence point for all three complement pathways and the most abundant complement protein, complement component 3 (C3) is implicated as a driver of acute inflammation after ICH, and can exacerbate the oxidative stress response, neuronal cell death, and brain injury.^8^, ^9^ Moreover, several previous studies have shown that increased serum complement C3 levels are associated with brain injury after cerebral ischemia and C3‐deficient mice exhibit decreased microglial activation, reduced neutrophil infiltration, and brain edema following ICH.^10^, ^11^ Thus, complement C3 plays a pivotal role in brain injury after ICH, but the plasma C3 levels in the peripheral blood of ICH patients and the mechanism through C3 affects brain injury in ICH patients have not been fully elucidated.

Synaptic pruning is a natural process in which microglia selectively engulf redundant synapses to form correct neural circuits during the development of the central nervous system (CNS).^12^ As tissue‐resident macrophages of the CNS, microglia play an important role in the monitoring and intervention of synaptic plasticity.^13^ Previous studies have shown increased phagocytic activity of microglia in the brain in various neurological diseases, and mounting evidence shows that microglia mediate synaptic elimination in a complement‐ and activity‐dependent manner. Moreover, depletion of microglia or inhibition of the complement cascade reduces the extent of synapse loss.^14^, ^15^ Shi et al.^16^ demonstrated that microglia are involved in engulfing synapses in the region of reactive gliosis and that inhibiting phagocytosis of microglia attenuated synapse loss and improved neurobehavioral outcomes in hemorrhagic stroke model mice. Another study revealed that activation of complement‐ and microglia‐dependent synaptic pruning leads to a decrease in synaptic density and subsequent cognitive decline in ischaemic stroke model mice. Importantly, complement inhibition effectively reduces microgliosis and synaptic uptake, and improves cognitive outcomes.^17^ Taken together, these findings suggest that microglia mediate synaptic pruning following ICH through the complement system, but the specific complement molecules involved are still unclear and need further investigation.

The brain is highly sensitive to hypoxia, and normobaric oxygen (NBO) therapy, which delivers high‐flow oxygen through a facemask at normobaric pressure, has been regarded as a neuroprotective treatment for ischemic stroke.^18^ Previous clinical studies by our laboratory and others have confirmed the safety and efficacy of NBO therapy for ICH‐mediated brain damage.^19^, ^20^, ^21^ A previous study reported that early NBO treatment slows blood‐brain barrier damage and improves neurological outcomes after cerebral ischemia.^22^ Another experimental study reported that NBO plays a neuroprotective role after cerebral ischemia by activating the Nrf2/HO‐1 antioxidative stress pathway.^23^ Moreover, in a collagenase‐induced rat model, NBO therapy strongly ameliorated brain edema and reduced neurological function defects after ICH.^24^ These studies demonstrated the neuroprotective role of NBO in ICH‐mediated brain damage, but the possible underlying mechanism remains elusive. Therefore, we hypothesized that NBO may play a neuroprotective role by reducing the level of C3 in microglia and alleviating microglia‐mediated synaptic pruning after ICH.

In the present study, we measured plasma C3 levels in ICH patients and controls, evaluated its correlation with hemorrhagic severity and clinical outcomes, confirmed the neuroprotective function of NBO therapy in hemorrhagic damage, and further investigated the mechanism of NBO therapy in C3‐mediated brain injury after ICH.

## 2 METHODS

### 2.1 Peripheral blood sample collection

Acute spontaneous ICH patients who were diagnosed via head computed tomography (CT) (GE 256‐row ultrahigh‐end spiral CT, USA) scans at the Affiliated Hospital of Jiujiang University (Jiujiang, China) from January 2021 to May 2022 were enrolled in this retrospective study. All patients were hospitalized within 24 h after stroke, and their hematomas were treated nonoperatively. The matched healthy controls were selected from patients that visited our medical examination center between January 2022 and May 2022. The inclusion and exclusion criteria have been previously described.^25^ Peripheral blood samples were centrifuged at 3000 *g* for 10 min, separated and immediately preserved at −80°C for subsequent analysis. Written informed consent was obtained from the patients or their relatives and the control individuals, and the study was approved by the Medical Ethics Committee of the Affiliated Hospital of Jiujiang University (Grant No. IRB2022‐JJU‐032‐21).

### 2.2 Clinical outcome assessment and plasma C3 level determination

Disease severity was assessed using the National Institutes of Health Stroke Scale (NIHSS) score and the Glasgow Coma Scale (GCS) score.^26^, ^27^ Plasma C3 levels were quantitatively measured with a Coulter reagent according to the manufacturer's instructions on the Beckman Coulter AU5800 clinical chemistry analyzer (Beckman Coulter Inc., California, USA). The test was performed and analyzed by the same technician.

### 2.3 Clinical image collection and evaluation

The CT scans of each patient were taken and semiautomatically analyzed for assessment of hematoma and perihemorrhagic edema volumes using Siemens Leonardo V software. CT perfusion (CTP) maps of cerebral blood flow (CBF), cerebral blood volume (CBV), mean transition time (MTT) and time to peak (TTP) were used to evaluate cerebral perfusion.

### 2.4 ICH mouse model construction

Sixty 18‐month‐old (25–30 g) male C57BL/6 mice were obtained from the Animal Center of Hubei Province. The collagenase‐induced ICH model was generated as previously described.^28^ Briefly, mice were anesthetized with pentobarbital sodium (40 mg/kg, 1%, intraperitoneal) and immobilized on a stereotaxic apparatus (RWD Life Science Co., Shenzhen, China). A total of 0.075 U of collagenase VII (Sigma Aldrich) dissolved in 5 μL of saline was microinfused into the ICH model mice (anterior: 0.2 mm, lateral: 3 mm, ventral: 4 mm) via a cannula connected to a Hamilton microsyringe pump (WPI, Sarasota, FL). The sham group was injected with an equal volume of saline at the same intracranial location. All of the experiments were approved and supervised by the Medical Ethics Committee at the Affiliated Hospital of Jiujiang University.

### 2.5 Normobaric hyperoxia (NBO) treatment

A total of 16 hospitalized patients who met the inclusion criteria were immediately and randomly assigned to the ICH or ICH + NBO group. The mice were randomly assigned to three groups—the Sham group, the ICH group and the ICH + NBO group—for which the researchers were blinded. NBO therapy was administered according to our previous study.^19^ In brief, patients in the NBO group were given high‐flow (8 L/min) mask oxygen for 1 h and intermittent periods at a flow rate of 2 L/min 6 times daily, and the control patients were given low‐flow mask oxygen (2 L/min, daily). Similarly, after 1 h of recovery from anesthesia, the ICH model mice were exposed to a hyperoxic box with 90% oxygen (5 L/min) for 1 h, 6 times daily, and the control mice were exposed to a paired airflow.

### 2.6 Behavioral tests

Behavioral tests were performed at 7 days after collagenase‐induced hemorrhage using the neurological deficit score, forelimb placing test, corner turn test and Morris water maze test, according to previous studies.^29^, ^30^

Motor and sensory deficits following injury were scored using a 28‐point neurological scoring system, in which a higher score indicates a more severe injury.

For the forelimb placing test, each mouse was tested 10 times, and the percentage of trials in which the mouse placed the appropriate forelimb on the edge of the countertop in response to the vibrissae stimulation was recorded.

For the corner turn test, the mice were placed in a corner at an angle of 30°. Their choice of turn direction was noted, and the number of right or left turns out of 10 total attempts was recorded.

For the Morris water maze test, the mice were trained to find a hidden platform (1 cm under water) using a series of cues on the walls, four trials per day for 5 consecutive days. The swimming path and the time used to locate the platform (latency) were recorded by a camera connected to a digital tracking device attached to an IBM computer. On Day 9, the hidden platform was removed, and the swimming path and latency time were recorded. These tests were performed by investigators blinded to the experimental group assignments.

### 2.7 Immunofluorescence staining

Mice were euthanized and immediately perfused with phosphate‐buffered saline (PBS) and 4% paraformaldehyde (PFA) solution. The brain tissues were dissected and fixed at 4°C for 24 h, and brain sections (20 μm) were prepared as described previously.^31^ Free‐floating brain sections were permeabilized with 0.5% Triton X‐100 in PBS for 30 min and washed with PBS 3 times. After blocking with 3% bovine serum albumin (BSA) for 30 min at room temperature, the sections were incubated with primary antibodies against Iba1 (diluted 1:500; Wako, 019‐19741) and C3 (diluted 1:500; ab11862, Abcam) at 4°C overnight. After washing with PBS 3 times, the sections were incubated with a secondary antibody at room temperature for 1 h. Finally, the sections were stained with DAPI (1:5000, Sigma) for 10 min and imaged using a fluorescence microscope (LSM800, Carl Zeiss, Germany).

### 2.8 Western blotting

Mice were decapitated and the proteins were separated as described previously.^32^ Equal amounts of protein were separated by 10% sodium dodecyl sulfate–polyacrylamide gels electrophoresis (SDS–PAGE) and transferred to nitrocellulose membranes. The membranes were blocked with 3% milk for 1 h at room temperature and then incubated with primary antibodies against SYP (diluted 1:800; ab52636, Abcam), PSD95 (diluted 1:800; ab238135, Abcam), NR2B (diluted 1:100; ab28373, Abcam), C3 (diluted 1:500; ab97462, Abcam) and β‐actin (diluted 1:1000; 66009‐1‐Ig, Proteintech) overnight at 4°C. Subsequently, the blots were incubated with goat anti‐mouse or anti‐rabbit antibodies conjugated to the IRdye 800 secondary antibody for 1 h at room temperature, after which the membranes were washed 5 times with TBST for 10 min each time. The protein bands were visualized and quantified by an Odyssey Imaging System (LI‐COR, Lincoln, NE, USA).

### 2.9 Transmission electron microscopy (TEM)

Mice were anesthetized and immediately perfused with PBS and 4% PFA solution. Then, the mice brains were fixed in 2% glutaraldehyde at 4°C overnight, and the perihemorrhagic tissues were dissected. Subsequently, the tissues were washed with cacodylate buffer, postfixed in 1% osmium tetroxide for 2 h at room temperature, washed with cacodylate buffer again, incubated in 1% uranyl acetate for 1 h and dehydrated in a graded series of ethanol. Finally, the tissues were infiltrated with propylene oxide and Epon series, sectioned with an ultramicrotome (Leica, Weztlar, Germany), and placed on electron microscope grids. Sections were stained with 2% uranyl acetate and 6% lead citrate and then observed under a Hitachi HT‐7700 (Japan) electron microscope.

### 2.10 Golgi staining

Mice were anesthetized and perfused with PBS, followed by 4% PFA solution. Thirty‐micron‐thick brain sections were cut with a vibrating microtome (Leica, Wetzlar, Germany). Golgi staining was performed using an FD Rapid Golgi Stain Kit (PK401) (FD NeuroTechnologies, USA) following the manufacturer's instructions. Images of dendritic spines were taken using a confocal microscope (Leica, DM6000B, Germany) and analyzed using an ImageJ software.

### 2.11 Statistical analysis

The data are shown as the mean ± standard error of the mean (mean ± SEM), and statistical analyses were performed with GraphPad Prism 9 (GraphPad Software, San Diego, CA, USA). Unpaired *t*‐tests or Mann–Whitney *U* tests were used to compare the differences between the two groups. The Kruskal–Wallis *H* test or one‐way or two‐way ANOVA and post hoc test were used for multiple comparisons. The Spearman correlation coefficient was calculated to analyze bivariate correlations, and logistic regression analysis was performed to assess the association between plasma C3 levels and clinical outcomes after ICH. *p* < 0.05 was considered to indicate statistical significance.

## 3 RESULTS

### 3.1 High plasma C3 levels were correlated with hemorrhagic severity and clinical outcomes in ICH patients

**FIGURE 1:**
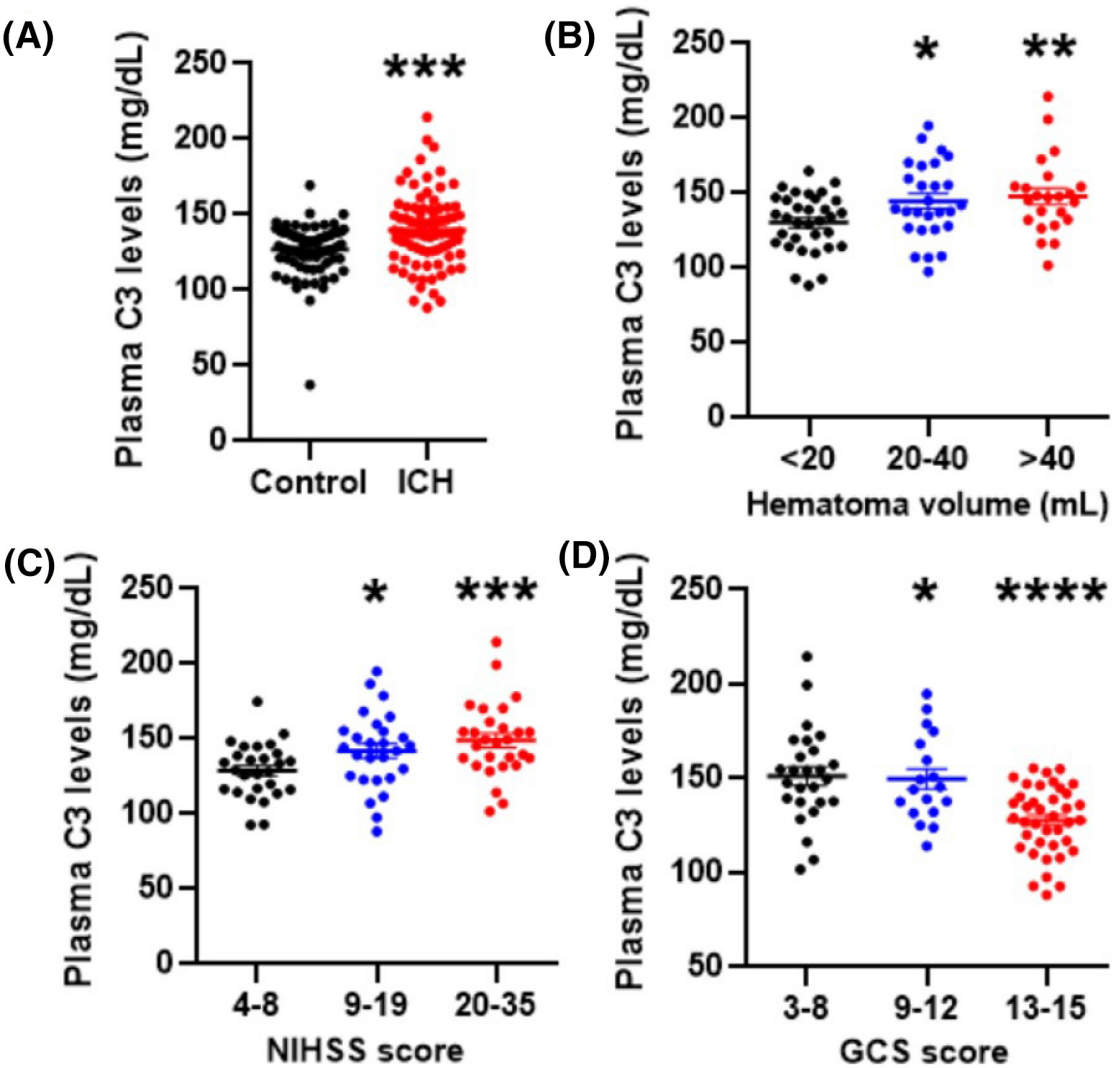
High plasma C3 levels and poor clinical outcomes after ICH. (A) The level of complement C3 in the plasm of ICH patients (*n* = 83) and control individuals (*n* = 78). (B–D) Association of plasma C3 levels with the hematoma volume (B), NIHSS score (C), and GCS score (D) in ICH patients. Statistical analyses were performed using the Mann–Whitney *U* test or the Kruskal–Wallis *H*‐test. *p < 0.05; **p < 0.01; ***p < 0.001; ****p < 0.0001. GSC, Glasgow Coma Scale; ICH, intracerebral hemorrhage; NIHSS, National Institutes of Health Stroke Scale.

We recruited 83 ICH patients and 78 healthy controls in this study. The demographic and clinical information about the participants is listed in Table S1. We first collected peripheral blood samples from patients and controls to measure the expression levels of C3. The plasma C3 levels in ICH patients were significantly increased compared with those in healthy controls (139.9 ± 2.655 vs. 126.8 ± 1.900, *p* < 0.0001) (Figure 1A), which indicated that C3 participates in the progression of ICH. We further investigated the relationship between plasma C3 levels and hemorrhagic severity, as indicated by hematoma volume, NIHSS score and GCS score, in this cohort of ICH patients. Interestingly, we found that the larger the hematoma volume and NIHSS score were, the higher the plasma C3 level was (Figure 1B,C). Similarly, compared to patients with higher GCSs, those with lower GCSs had higher plasma C3 levels (Figure 1D). Therefore, hemorrhagic injury upregulates plasma C3 expression in ICH patients, and plasma C3 levels are closely related to hemorrhagic severity.

**FIGURE 2:**
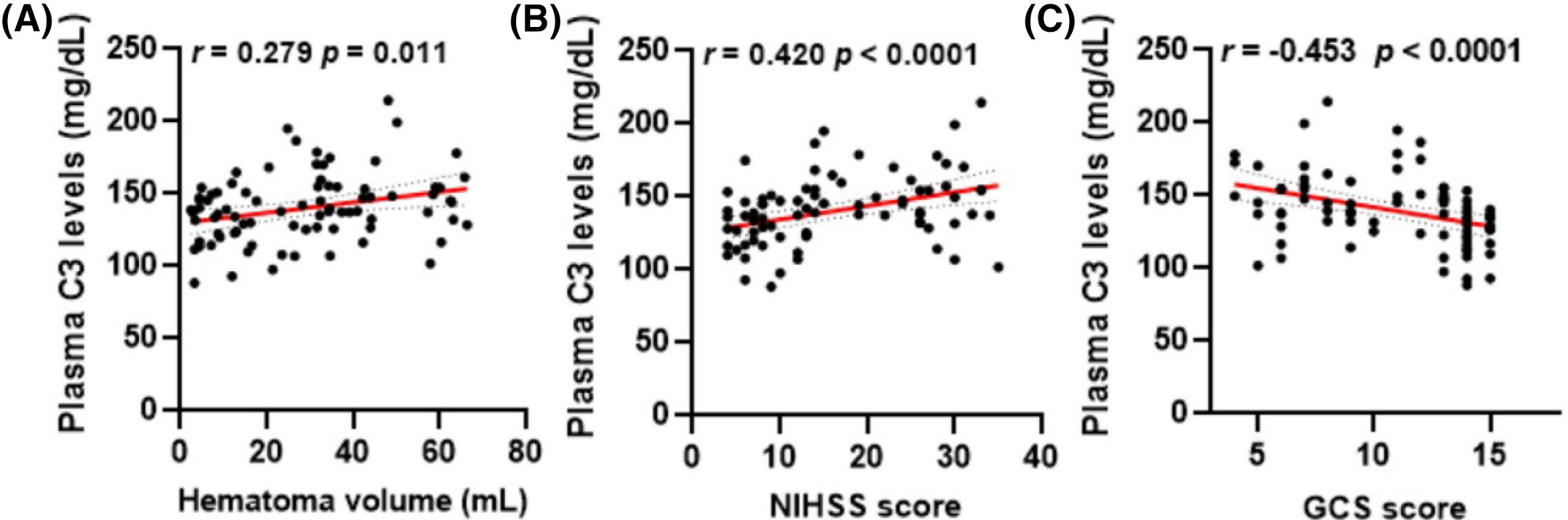
Plasma C3 levels were strongly correlated with neurologic functional outcomes. (A–C) Correlation analysis between plasma C3 levels and hematoma volume (A), NIHSS score (B), and GCS score (C) in ICH patients (*n* = 83). Statistical analyses were performed using Spearman's correlation coefficient.

We then used Spearman's correlation coefficient to verify the correlations between plasma C3 levels and hematoma volume, NIHSS score, and GCS score in these patients. Our findings revealed that plasma C3 levels are positively correlated with hematoma volume (*r* = 0.279, *p* < 0.05) and the NIHSS score (*r* = 0.42, *p* < 0.0001), but are negatively correlated with the GCS score (*r* = −0.453, *p* < 0.0001) after ICH (Figure 2). Moreover, patients with high plasma C3 levels had larger hematoma volumes, higher NIHSS scores, and lower GCS scores than those with high plasma C3 levels according to multiple logistic regression analysis (Table S2), indicating poor clinical outcomes after ICH. Taken together, these results demonstrated that plasma C3 levels are strongly correlated with disease severity and ICH patient outcomes.

### 3.2 NBO treatment improved neurological deficits and downregulated the expression of C3 after ICH in patients and model mice

**FIGURE 3:**
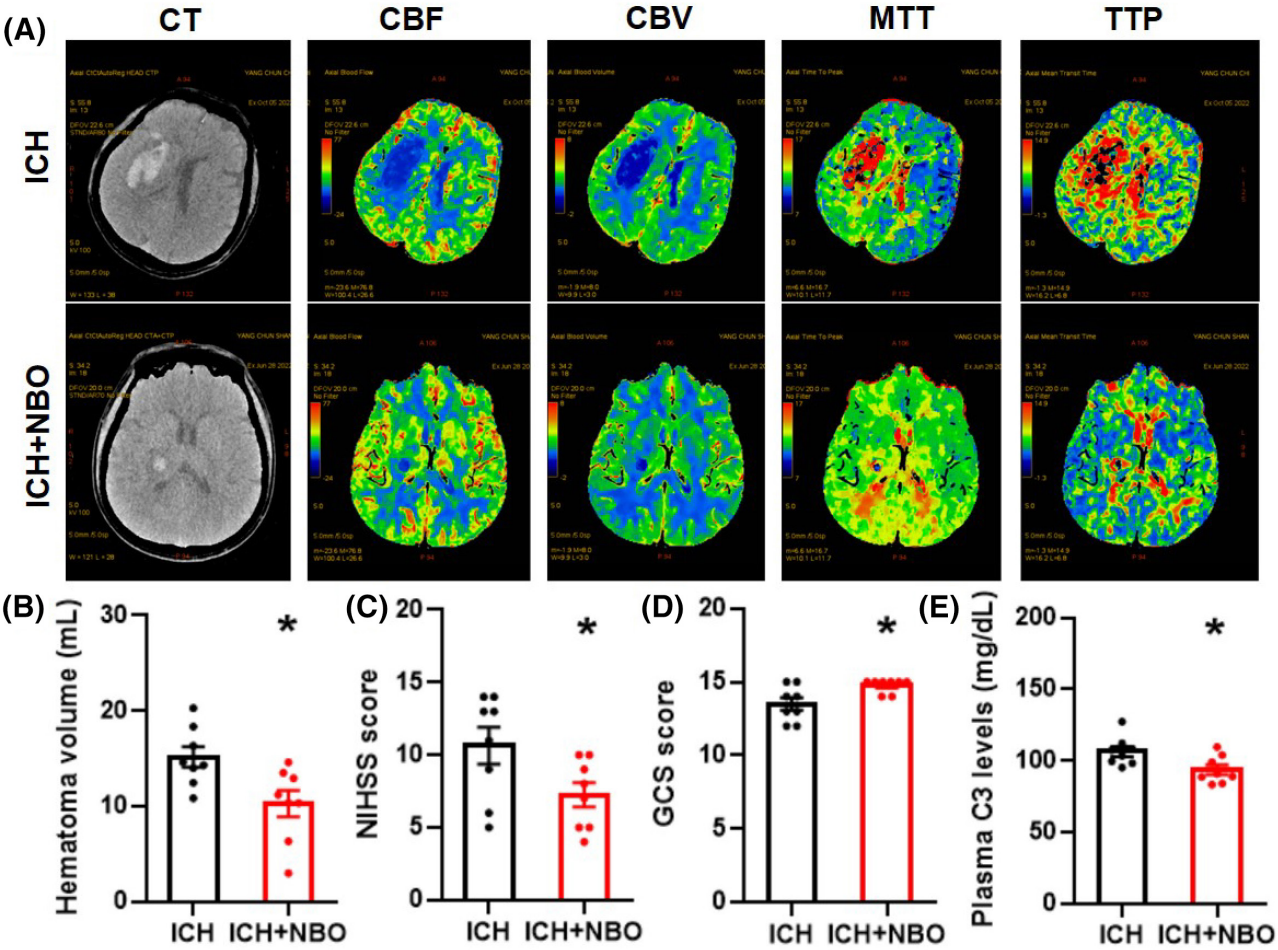
NBO therapy improved overall neurological function and reduced plasma C3 levels in ICH patients after 7 days. (A) Representative CT and CTP images at 7 days post‐ICH in the control (ICH) and NBO (ICH + NBO) groups. (B–E) Hematoma volume (B), NIHSS score (C), GCS score (D) and plasma C3 level (E) after 7 days in the control and NBO groups. CBF, cerebral blood flow; CBV, cerebral blood volume; CT, computed tomography; CTP, computed tomography perfusion; MTT, mean transition time; NBO, normobaric hyperoxia; TTP, time to peak. *n* = 8 per group, and statistical analyses were performed using the *t* test; *p < 0.05.

To determine whether NBO is involved in complement C3‐mediated brain injury after ICH, we treated ICH patients with (ICH + NBO) or without (ICH) NBO therapy and examined plasma C3 levels and subsequent neurological functions. There was no significant difference in the baseline hematoma volume between the ICH group and the ICH + NBO group (Figure S1). Figure 3A shows representative clinical images of the patients in the two groups. NBO treatment obviously improved cerebral perfusion (Figure 3A) and markedly reduced the hematoma volume and NIHSS score (Figure 3B,C). Moreover, NBO treatment also effectively increased the GCS score (Figure 3D), and significantly decreased the plasma C3 levels in ICH patients (Figure 3E). This clinical evidence demonstrated the effects of NBO therapy on plasma C3 levels and hemorrhagic‐induced brain damage.

**FIGURE 4:**
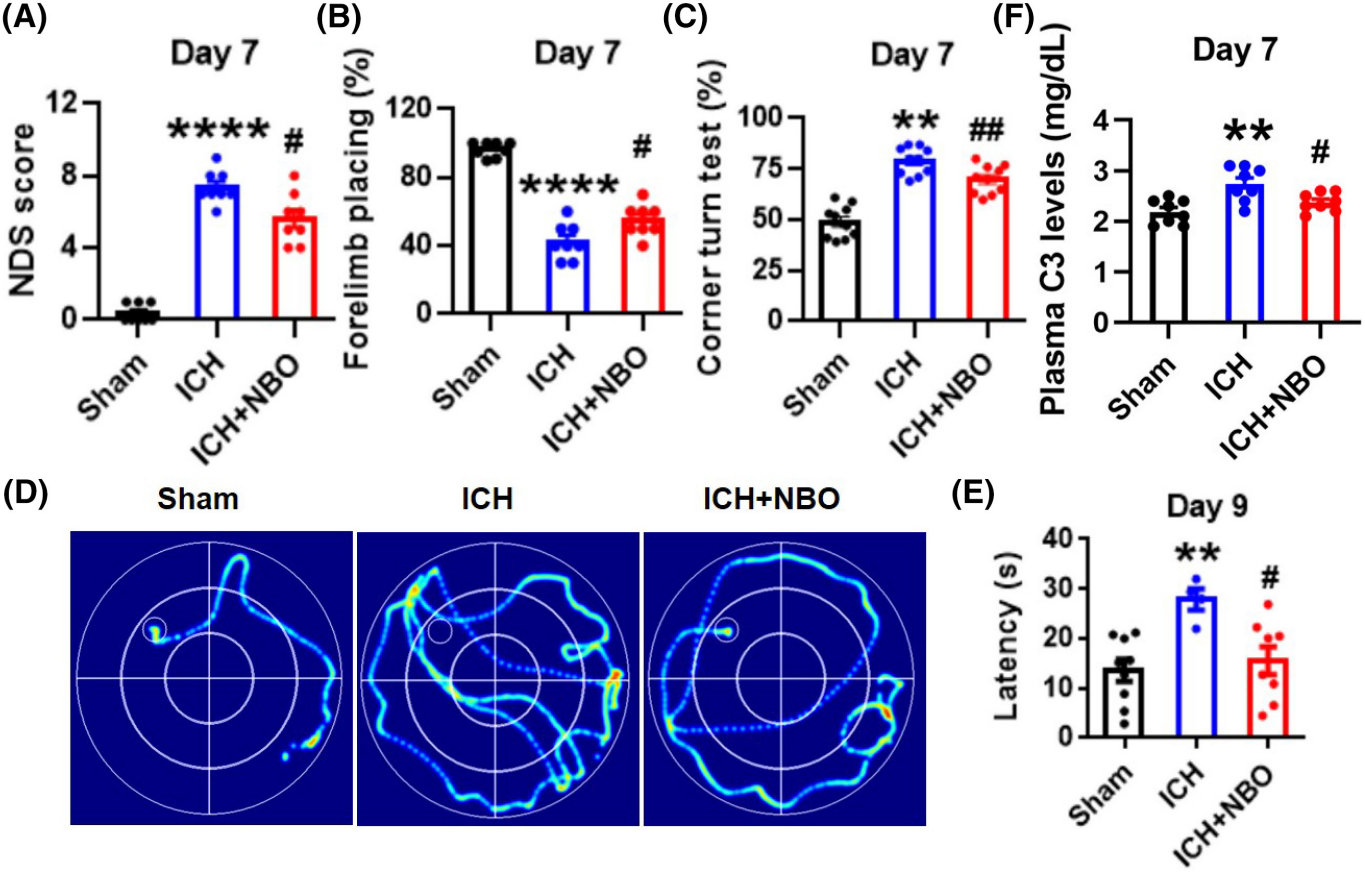
NBO therapy protected against hemorrhagic‐induced brain damage and C3 upregulation in vivo. (A–C) At 7 days after ICH, the neurologic deficit score (A), forelimb placement score (B) and corner turn test (C) were evaluated. (D, E) In the Morris water maze, representative searching traces on Day 7 (D) and the latency to reach the hidden platform on Day 9 (E) were recorded. (F) Plasma C3 levels at 7 days after ICH. The data are presented as the means ± SEMs, *n* = 8 per group. **p < 0.01, ****p < 0.0001 versus the sham group; #*p* < 0.05, ##*p* < 0.01 versus the ICH group; one‐way ANOVA with Tukey's multiple comparison test.

To further confirm the neuroprotective function of NBO therapy in ICH, we established a collagenase‐induced mouse model (Figure S2). Figure S3 shows the experimental design, including the NBO treatment, ICH construction, behavioral test, tissue collection and neuronal injury assessment. The neurological deficit score, forelimb‐placement test, corner turn test and Morris water maze test were performed at 7 days after ICH to evaluate the neurological outcomes. As expected, the behavioral test results showed that ICH model mice had increased NDS scores (Figure 4A), more severe forelimb muscle weakness (Figure 4B), fewer correct corner turns (Figure 4C) and longer latency to reach the hidden platform (Figure 4D,E). Importantly, NBO treatment obviously alleviated all of these neurologic deficits and downregulated the expression of C3 in ICH model mice (Figure 4F). These data clearly showed that NBO treatment attenuates the upregulation of C3 after ICH and plays a neuroprotective role in both ICH patients and model mice.

### 3.3 NBO treatment protected against hemorrhagic‐induced brain damage by inhibiting the expression of C3 in microglia

**FIGURE 5:**
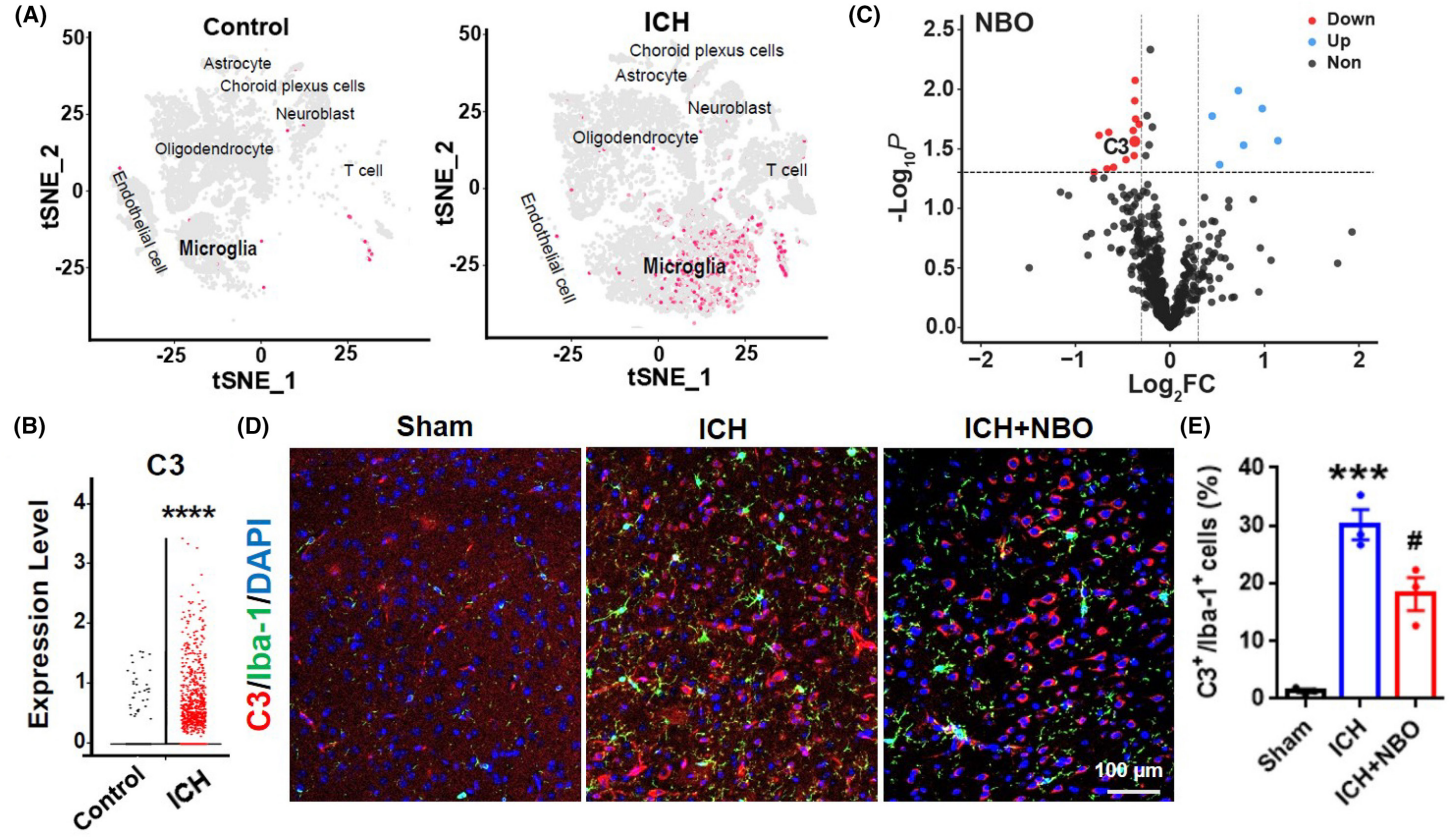
NBO therapy inhibited the expression of C3 in microglia after ICH. (A) A tSNE map from the scRNA‐seq dataset GSE167593 shows the C3 mRNA expression profiles of the mice in the control and ICH groups. The red dots represent the mRNA expression levels of the C3 gene in different cell subtypes. (B) Expression levels of C3 in microglia in the different groups. (C) Proteomic mass spectrometry revealed the differentially expressed proteins in the plasma of NBO‐treated patients. (D, E) Representative images and quantitative analysis of immunofluorescence staining for C3 (red) and Iba‐1 (green) in the different groups. DAPI, blue; scale bars = 100 μm. ***p < 0.001, ****p < 0.0001 versus sham group; ^#^ *p* < 0.05 versus ICH group; *t* test.

**FIGURE 6:**
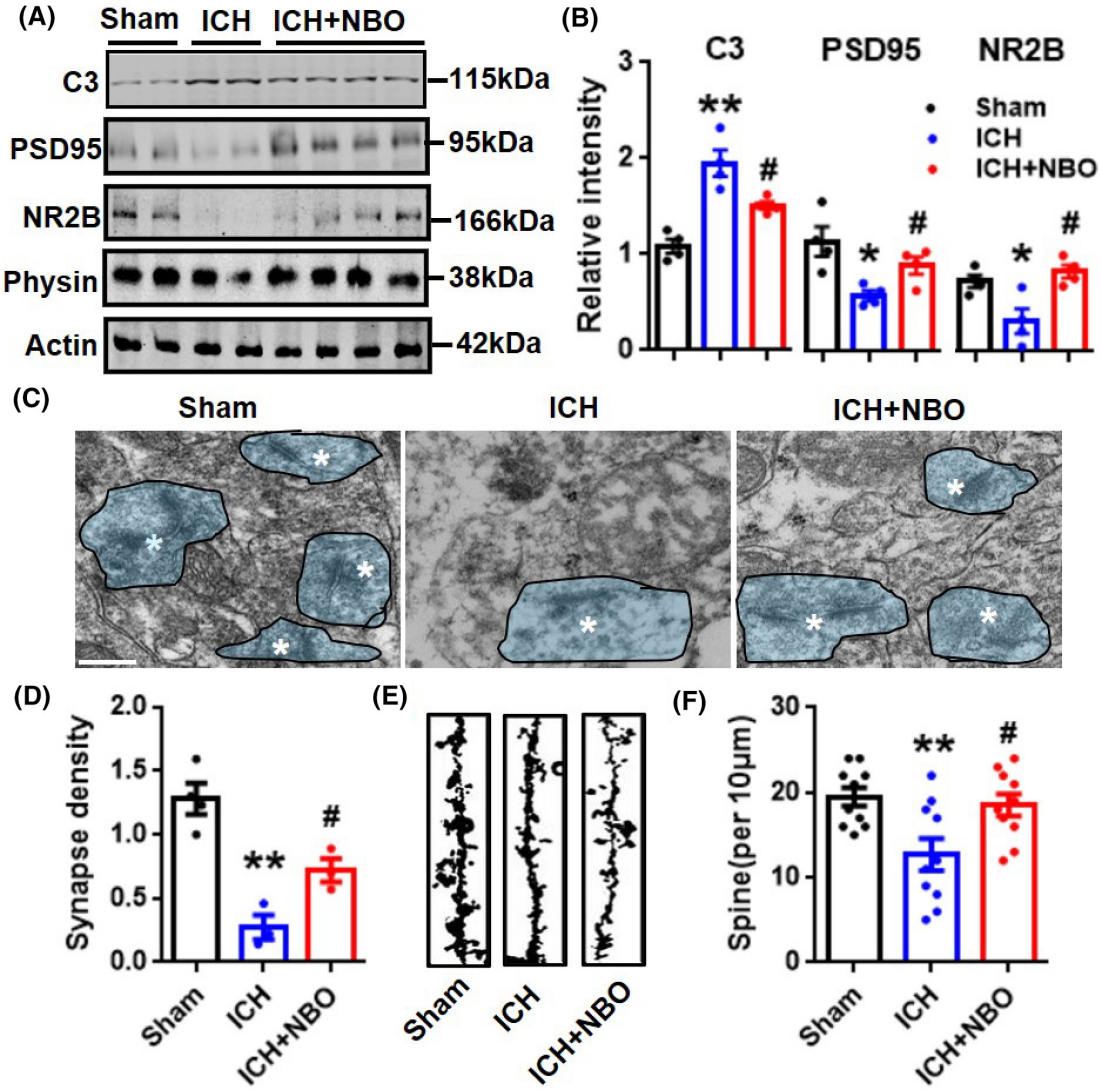
NBO therapy alleviates microglia‐mediated synaptic pruning and brain injury after ICH. (A, B) Representative blots (A) and quantitative analysis (B) of C3, PSD95, Physin and NR2B levels in the different groups. *n* = 4 mice per group. C3, complement 3; PSD95, postsynaptic density protein 95; Physin, synaptophysin; NR2B, N‐methyl‐D‐aspartate (NMDA) receptor type 2B. (C, D) Representative TEM images of the synaptic ultrastructure in the perihematoma (C) and quantitative analysis of synapse density (blue, indicated by asterisks) (D) are presented. Scale bars = 500 nm. *n* = 4 mice per group. (E, F) Representative Golgi staining images (E) are shown, and quantitative analyses of spine density (F) were performed. 10 sections from each group were randomly chosen for statistical analysis; *n* = 3 mice per group. Scale bars = 5 μm. The data are presented as the mean ± SEM. *p < 0.05, **p < 0.01, versus sham group; #*p* < 0.05 versus ICH group; one‐way ANOVA with Tukey's multiple comparison tests.

Next, we explored the probable mechanisms by which NBO protects the brain against C3‐mediated brain injury after ICH. We then analyzed publicly available single‐cell RNA sequencing data (GSE167593) to determine the specific cell‐type mRNA expression levels of C3 after ICH^16^. A t‐distributed stochastic neighbor embedding (tSNE) map revealed various cell types associated with C3 activation after ICH, including oligodendrocytes, microglia, T cells and endothelial cells. In particular, the single‐cell RNA‐seq data revealed that C3 mRNA is expressed mainly in microglia, rarely expressed in astrocytes or neurons, and the C3 mRNA level in microglia obviously increased after ICH (Figure 5A). Moreover, statistical analysis showed that hemorrhagic injury significantly upregulated the expression of C3 in the microglia when compared with that in the microglia of controls (Figure 5B), and proteomic mass spectrometry revealed marked downregulation of C3 in NBO‐treated patients (Figure 5C). Costaining revealed that C3 was expressed mainly on inflammatory microglia but not on astrocytes in the perihematomal tissue 7 days after ICH. This finding is consistent with previous studies showing that C3 is expressed mainly in microglia.^15^, ^33^ Moreover, immunofluorescence staining and quantitative analysis revealed that C3 levels in the tissues of control mice were very low but were significantly increased and mostly colocalized with IBA1‐positive cells in ICH model mice; the ICH‐mediated upregulation of C3 in microglia was inhibition by NBO treatment (Figure 5D,E). In addition, the C3 protein level was significant increased in ICH model mice compared with sham mice, and NBO treatment effectively prevented these increases, as determined by Western blotting (Figure 6A,B). Moreover, complement C3 plays a critical role in ICH‐induced brain injury and targeted C3 inhibition improves neurological outcomes following ICH.^10^, ^11^ Taken together, these results suggested that the upregulation of C3 in microglia mediates brain injury after ICH and that NBO therapy protects against brain damage by attenuating the activation of C3 in microglia.

### 3.4 NBO therapy attenuated microglia‐mediated synaptic pruning after ICH

Microglia‐mediated synaptic pruning plays a prominent role in hemorrhagic‐induced brain damage.^34^ Finally, we investigated the effect of NBO therapy on the synaptic loss after ICH. Western blotting revealed that ICH significantly decreased the expression levels of postsynaptic proteins (PSD95 and NR2B) in mice, but had no effect on the expression of the presynaptic marker synaptophysin. However, NBO treatment effectively restored the expression levels of PSD95 and NR2B (Figure 6A,B). Further TEM analysis confirmed the dramatically reduced synapse density, which was markedly increased in NBO‐treated mice (Figure 6C,D). Golgi staining also revealed synaptic loss after ICH, and dendritic spine deterioration was ameliorated after NBO treatment (Figure 6E,F). Previous study demonstrated that more than 50% of microglia and only 5% of astrocytes are phagocytic and that few synapses are engulfed by astrocytes in collagenase‐induced ICH mice model.^16^ Our gene score feature plots results indicated that the synaptic pruning pathway of microglia was overactive, while that of astrocytes did not change after ICH (Figure S4). Together, these results indicated that C3 activation promotes microglia‐dependent synaptic loss and that NBO therapy effectively reduces the level of C3 and rescues microglia‐mediated synaptic pruning after ICH.

## 4 DISCUSSION

Previous studies have reported the vital role of complement C3 in brain injury after experimental ICH and ischaemic stroke,^8^, ^10^, ^35^ but the role of C3 in hemorrhagic stroke has not been determined. In the present study, we observed that plasma C3 levels increased as a result of hemorrhagic stroke, not only in ICH patients but also in hemorrhagic mice. Moreover, we also found that plasma C3 levels were closely related to hemorrhagic severity and neurologic functional outcomes after ICH. Interestingly, a previous clinical study reported that the serum complement C3 concentration is a valuable prognostic biomarker of ischaemic stroke.^8^ Thus, these finding are consistent with our data revealing that plasma C3 levels can be used as an effective biomarker of clinical outcomes in both ischaemic and hemorrhagic stroke patients. Moreover, brain damage following ICH is often attributed to microglial activation and polarization.^36^ Here, we reported that hemorrhagic stroke led to the activation of microglia and a remarkable increase in C3 expression levels in microglia, whereas NBO treatment significantly reduced C3 levels, which reflects decreased brain damage in these patients and animals. Collectively, our data not only provide direct evidence for the involvement of plasma C3 levels in brain injury after hemorrhagic stroke but also suggest a new therapeutic approach for this disease.

NBO plays a neuroprotective role against traumatic brain injury (TBI) and stroke,^37^, ^38^ but the relevant mechanisms involved in this process have not been elucidated. In this study, our clinical and experimental results demonstrated that NBO treatment markedly reduces neurological deficits and brain damage following ICH in both patients and animals, confirming the neuroprotective functions of NBO after ICH. Moreover, we also found that NBO alleviates brain injury following ICH by downregulating the expression of C3 in microglia and the inhibiting complement system activation. Previous efforts to inhibit complement system activation have focused mainly on genetic deletion or monoclonal antibody neutralization,^39^, ^40^ but while these approaches are suitable for experimental research, they are not convenient for clinical use. This study is the first to report that NBO therapy, which is safety and easy to use in clinical practice, inhibits complement activation. Since overactivation of the complement system and C3‐mediated brain injury are common in animals and humans, our findings could lead to the use of a potentially universal strategy for treating several neurological disorders, including Alzheimer's disease (AD), TBI, epilepsy and depression.^41^, ^42^, ^43^, ^44^

Complement activation mediates microglial synapse elimination after stroke,^45^, ^46^ but the specific complement molecule that regulates microglia‐mediated synaptic loss following ICH has not yet been identified. After ICH, the expression of C3 was upregulated, and the expression of postsynaptic proteins and dendritic spines was reduced. When NBO therapy was used, the activation of C3 in microglia was suppressed, and the synaptic indices were restored, which ultimately lead to the mitigation of synaptic loss and neurological deficits in patients and animals. Notably, markedly increased expression levels of C3 and C3‐mediated synaptic elimination have been reported in multiple neurological disorders, such as AD, multiple sclerosis (MS), schizophrenia (SCZ), frontotemporal dementia (FTD) and otherneurodegenerative diseases.^46^, ^47^, ^48^ Thus, our results revealed a previously unidentified role for C3‐mediated synaptic elimination in brain injury following hemorrhagic stroke. NBO is a very common clinical adjuvant treatment, and the possiblemechanisms of NBO therapy include reducing blood occludin fragments, suppressing oxidative stress and improving mitochondrial function.^21^, ^23^, ^49^ Importantly, our results revealed that NBO alleviates brain injury after ICH by inhibiting complement system activation and attenuating C3‐mediated synaptic pruning. Therefore, we also revealed a novel protective mechanism of NBO therapy.

In summary, our findings demonstrated that NBO plays a neuroprotective role by reducing the level of C3 in microglia and alleviating microglia‐mediated synaptic pruning, suggesting that NBO therapy might be an effective clinical treatment for ICH patients and targeting microglial C3 may be a potential therapeutic approach for treating neurological disorders caused excessive synaptic elimination.

## AUTHOR CONTRIBUTIONS

MXW, XPY and ZYC were responsible for conceiving and designing the study. MXW, KC and WMY developed the method, performed the data visualization and statistical analysis, and prepared the manuscript. YSZ, MJ and BB acquired, collected, and extracted the data included in this analysis. MXW, WMY and KC analyzed the data. ZYC, MJ and YSZ helped with manuscript preparation and data review. The final version of the manuscript was approved by all of the authors. All of the authors contributed to the article and approved the submitted version.

## FUNDING INFORMATION

This study was supported partially by the National Natural Science Foundation of China (82260209 and 81960221 to XPY and 82203926 to KC); the Jiangxi Provincial Natural Science Foundation of China (20232BAB206046 to MXW); the Hubei Provincial Natural Science Foundation of China (2022CFB955 to KC); the Science and Technology Project Founded by the Education Department of Jiangxi Province (GJJ201834 to MXW); and the Jiangxi Provincial Health Commission Science and Technology Plan project (202212021 to MXW and 202311506 to ZYC).

## CONFLICT OF INTEREST STATEMENT

The authors declare that they have no conflicts of interest.

## Supporting information


Appendix S1

## Data Availability

The data that support the findings of this study are available from the corresponding author upon reasonable request.

## References

[1] Peng C , Wang Y , Hu Z , Chen C . Selective HDAC6 inhibition protects against blood‐brain barrier dysfunction after intracerebral hemorrhage. CNS Neurosci Ther. 2023;30:e14429. 37665135 10.1111/cns.14429 PMC10915991

[2] Zille M , Farr TD , Keep RF , Römer C , Xi G , Boltze J . Novel targets, treatments, and advanced models for intracerebral haemorrhage. EBioMedicine. 2022;76:103880. 35158309 10.1016/j.ebiom.2022.103880 PMC8850756

[3] Hemphill JC 3rd , Greenberg SM , Anderson CS , et al. Guidelines for the Management of Spontaneous Intracerebral Hemorrhage: a guideline for healthcare professionals from the American Heart Association/American Stroke Association. Stroke. 2015;46(7):2032‐2060. 26022637 10.1161/STR.0000000000000069

[4] Broderick JP , Grotta JC , Naidech AM , et al. The story of intracerebral hemorrhage: from recalcitrant to treatable disease. Stroke. 2021;52(5):1905‐1914. 33827245 10.1161/STROKEAHA.121.033484 PMC8085038

[5] Wu S , Wu B , Liu M , et al. Stroke in China: advances and challenges in epidemiology, prevention, and management. Lancet Neurol. 2019;18(4):394‐405. 30878104 10.1016/S1474-4422(18)30500-3

[6] Mastellos DC , Hajishengallis G , Lambris JD . A guide to complement biology, pathology and therapeutic opportunity. Nat Rev Immunol. 2023;24:118‐141. 37670180 10.1038/s41577-023-00926-1

[7] Holste K , Xia F , Garton HJL , et al. The role of complement in brain injury following intracerebral hemorrhage: a review. Exp Neurol. 2021;340:113654. 33617886 10.1016/j.expneurol.2021.113654 PMC8119338

[8] Yang P , Zhu Z , Zang Y , et al. Increased serum complement C3 levels are associated with adverse clinical outcomes after ischemic stroke. Stroke. 2021;52(3):868‐877. 33517703 10.1161/STROKEAHA.120.031715

[9] Geisbrecht BV , Lambris JD , Gros P . Complement component C3: a structural perspective and potential therapeutic implications. Semin Immunol. 2022;59:101627. 35760703 10.1016/j.smim.2022.101627 PMC9842190

[10] Wang M , Xia F , Wan S , Hua Y , Keep RF , Xi G . Role of complement component 3 in early erythrolysis in the hematoma after experimental intracerebral hemorrhage. Stroke. 2021;52(8):2649‐2660. 34176310 10.1161/STROKEAHA.121.034372 PMC8316397

[11] Yang S , Nakamura T , Hua Y , et al. The role of complement C3 in intracerebral hemorrhage‐induced brain injury. J Cereb Blood Flow Metab. 2006;26(12):1490‐1495. 16552422 10.1038/sj.jcbfm.9600305

[12] Morizawa YM , Matsumoto M , Nakashima Y , et al. Synaptic pruning through glial synapse engulfment upon motor learning. Nat Neurosci. 2022;25(11):1458‐1469. 36319770 10.1038/s41593-022-01184-5

[13] Paolicelli RC , Bolasco G , Pagani F , et al. Synaptic pruning by microglia is necessary for normal brain development. Science. 2011;333(6048):1456‐1458. 21778362 10.1126/science.1202529

[14] Wang C , Yue H , Hu Z , et al. Microglia mediate forgetting via complement‐dependent synaptic elimination. Science. 2020;367(6478):688‐694. 32029629 10.1126/science.aaz2288

[15] Hong S , Beja‐Glasser VF , Nfonoyim BM , et al. Complement and microglia mediate early synapse loss in Alzheimer mouse models. Science. 2016;352(6286):712‐716. 27033548 10.1126/science.aad8373 PMC5094372

[16] Shi X , Luo L , Wang J , et al. Stroke subtype‐dependent synapse elimination by reactive gliosis in mice. Nat Commun. 2021;12(1):6943. 34836962 10.1038/s41467-021-27248-x PMC8626497

[17] Alawieh AM , Langley EF , Feng W , Spiotta AM , Tomlinson S . Complement‐dependent synaptic uptake and cognitive decline after stroke and reperfusion therapy. J Neurosci. 2020;40(20):4042‐4058. 32291326 10.1523/JNEUROSCI.2462-19.2020 PMC7219298

[18] Qi Z , Yuan S , Liu KJ , Ji X . Normobaric hyperoxia plays a neuroprotective role after cerebral ischemia by maintaining the redox homeostasis and the level of connexin43 in astrocytes. CNS Neurosci Ther. 2022;28(10):1509‐1518. 35698913 10.1111/cns.13875 PMC9437237

[19] Chen Z , Ding J , Wu X , et al. Safety and efficacy of normobaric oxygenation on rescuing acute intracerebral hemorrhage‐mediated brain damage – a protocol of randomized controlled trial. Trials. 2021;22(1):93. 33499916 10.1186/s13063-021-05048-4 PMC7836205

[20] Li W , Qi Z , Ma Q , et al. Normobaric hyperoxia combined with endovascular treatment for patients with acute ischemic stroke: a randomized controlled clinical trial. Neurology. 2022;99(8):e824‐e834. 35715198 10.1212/WNL.0000000000200775

[21] Shi S , Qi Z , Ma Q , et al. Normobaric hyperoxia reduces blood occludin fragments in rats and patients with acute ischemic stroke. Stroke. 2017;48(10):2848‐2854. 28931617 10.1161/STROKEAHA.117.017713 PMC5659343

[22] Liang J , Qi Z , Liu W , et al. Normobaric hyperoxia slows blood‐brain barrier damage and expands the therapeutic time window for tissue‐type plasminogen activator treatment in cerebral ischemia. Stroke. 2015;46(5):1344‐1351. 25804925 10.1161/STROKEAHA.114.008599 PMC4414814

[23] Pei J , Cai S , Song S , et al. Normobaric hyperoxia plays a protective role against renal ischemia‐reperfusion injury by activating the Nrf2/HO‐1 signaling pathway. Biochem Biophys Res Commun. 2020;532(1):151‐158. 32838965 10.1016/j.bbrc.2020.07.004

[24] You P , Lin M , Li K , Ye X , Zheng J . Normobaric oxygen therapy inhibits HIF‐1α and VEGF expression in perihematoma and reduces neurological function defects. Neuroreport. 2016;27(5):329‐336. 26872098 10.1097/WNR.0000000000000542

[25] Wu M , Chen K , Jiang M , et al. High plasma complement C4 levels as a novel predictor of clinical outcome in intracerebral hemorrhage. Front Aging Neurosci. 2023;15:1103278. 36891553 10.3389/fnagi.2023.1103278 PMC9986541

[26] Currò CT , Fiume G , Cotroneo M , et al. Ischemic stroke and reperfusion therapies in diabetic patients. Neurol Sci. 2022;43(7):4335‐4348. 35146566 10.1007/s10072-022-05935-x

[27] Wan Y , Guo H , Bi R , et al. Clinical and prognostic characteristics of recurrent intracerebral hemorrhage: a contrast to first‐ever ICH. Front Aging Neurosci. 2022;14:860571. 35493945 10.3389/fnagi.2022.860571 PMC9047504

[28] Rynkowski MA , Kim GH , Komotar RJ , et al. A mouse model of intracerebral hemorrhage using autologous blood infusion. Nat Protoc. 2008;3(1):122‐128. 18193028 10.1038/nprot.2007.513

[29] Bao WD , Zhou XT , Zhou LT , et al. Targeting miR‐124/Ferroportin signaling ameliorated neuronal cell death through inhibiting apoptosis and ferroptosis in aged intracerebral hemorrhage murine model. Aging Cell. 2020;19(11):e13235. 33068460 10.1111/acel.13235 PMC7681046

[30] Bao WD , Pang P , Zhou XT , et al. Loss of ferroportin induces memory impairment by promoting ferroptosis in Alzheimer's disease. Cell Death Differ. 2021;28(5):1548‐1562. 33398092 10.1038/s41418-020-00685-9 PMC8166828

[31] Liu D , Tang H , Li XY , et al. Targeting the HDAC2/HNF‐4A/miR‐101b/AMPK pathway rescues tauopathy and dendritic abnormalities in Alzheimer's disease. Mol Ther. 2017;25(3):752‐764. 28202389 10.1016/j.ymthe.2017.01.018 PMC5363202

[32] Wang X , Liu D , Huang HZ , et al. A novel MicroRNA‐124/PTPN1 signal pathway mediates synaptic and memory deficits in Alzheimer's disease. Biol Psychiatry. 2018;83(5):395‐405. 28965984 10.1016/j.biopsych.2017.07.023

[33] Stevens B , Allen NJ , Vazquez LE , et al. The classical complement cascade mediates CNS synapse elimination. Cell. 2007;131(6):1164‐1178. 18083105 10.1016/j.cell.2007.10.036

[34] Zhou Y , Bhatt H , Mojica CA , et al. Mesenchymal‐derived extracellular vesicles enhance microglia‐mediated synapse remodeling after cortical injury in aging rhesus monkeys. J Neuroinflammation. 2023;20(1):201. 37660145 10.1186/s12974-023-02880-0 PMC10475204

[35] Zheng Y , Fan L , Xia S , et al. Role of complement C1q/C3‐CR3 signaling in brain injury after experimental intracerebral hemorrhage and the effect of minocycline treatment. Front Immunol. 2022;13:919444. 36189326 10.3389/fimmu.2022.919444 PMC9520460

[36] Lan X , Han X , Li Q , Yang QW , Wang J . Modulators of microglial activation and polarization after intracerebral haemorrhage. Nat Rev Neurol. 2017;13(7):420‐433. 28524175 10.1038/nrneurol.2017.69 PMC5575938

[37] Ejaz S , Emmrich JV , Sitnikov SL , et al. Normobaric hyperoxia markedly reduces brain damage and sensorimotor deficits following brief focal ischaemia. Brain. 2016;139(Pt 3):751‐764. 26767570 10.1093/brain/awv391

[38] Li Y , Lv W , Cheng G , et al. Effect of early normobaric hyperoxia on blast‐induced traumatic brain injury in rats. Neurochem Res. 2020;45(11):2723‐2731. 32902742 10.1007/s11064-020-03123-x

[39] Wu T , Dejanovic B , Gandham VD , et al. Complement C3 is activated in human AD brain and is required for neurodegeneration in mouse models of amyloidosis and tauopathy. Cell Rep. 2019;28(8):2111‐2123.e2116. 31433986 10.1016/j.celrep.2019.07.060

[40] Trzeciak A , Mongre RK , Kim MR , et al. Neutrophil heterogeneity in complement C1q expression associated with sepsis mortality. Front Immunol. 2022;13:965305. 35983035 10.3389/fimmu.2022.965305 PMC9380571

[41] Cornell J , Salinas S , Huang HY , Zhou M . Microglia regulation of synaptic plasticity and learning and memory. Neural Regen Res. 2022;17(4):705‐716. 34472455 10.4103/1673-5374.322423 PMC8530121

[42] Xie J , Cools L , Van Imschoot G , et al. *Helicobacter pylori*‐derived outer membrane vesicles contribute to Alzheimer's disease pathogenesis via C3‐C3aR signalling. J Extracell Vesicles. 2023;12(2):e12306. 36792546 10.1002/jev2.12306 PMC9931688

[43] Dong X , Fan J , Lin D , et al. Captopril alleviates epilepsy and cognitive impairment by attenuation of C3‐mediated inflammation and synaptic phagocytosis. J Neuroinflammation. 2022;19(1):226. 36104755 10.1186/s12974-022-02587-8 PMC9476304

[44] Wang J , Chen HS , Li HH , et al. Microglia‐dependent excessive synaptic pruning leads to cortical underconnectivity and behavioral abnormality following chronic social defeat stress in mice. Brain Behav Immun. 2023;109:23‐36. 36581303 10.1016/j.bbi.2022.12.019

[45] Stephan AH , Barres BA , Stevens B . The complement system: an unexpected role in synaptic pruning during development and disease. Annu Rev Neurosci. 2012;35:369‐389. 22715882 10.1146/annurev-neuro-061010-113810

[46] Cardozo PL , de Lima IBQ , Maciel EMA , Silva NC , Dobransky T , Ribeiro FM . Synaptic elimination in neurological disorders. Curr Neuropharmacol. 2019;17(11):1071‐1095. 31161981 10.2174/1570159X17666190603170511 PMC7052824

[47] Werneburg S , Jung J , Kunjamma RB , et al. Targeted complement inhibition at synapses prevents microglial synaptic engulfment and synapse loss in demyelinating disease. Immunity. 2020;52(1):167‐182.e167. 31883839 10.1016/j.immuni.2019.12.004 PMC6996144

[48] Zhou J , Wade SD , Graykowski D , et al. The neuronal pentraxin Nptx2 regulates complement activity and restrains microglia‐mediated synapse loss in neurodegeneration. Sci Transl Med. 2023;15(689):eadf0141. 36989373 10.1126/scitranslmed.adf0141 PMC10467038

[49] Dong W , Qi Z , Liang J , et al. Reduction of zinc accumulation in mitochondria contributes to decreased cerebral ischemic injury by normobaric hyperoxia treatment in an experimental stroke model. Exp Neurol. 2015;272:181‐189. 25891441 10.1016/j.expneurol.2015.04.005 PMC4609237

