# Supplementary material for: Normobaric hyperoxia alleviates complement C3‐mediated synaptic pruning and brain injury after intracerebral hemorrhage
Source: CNS Neurosci Ther. 2024 Mar 26;30(3):e14694. doi: 10.1111/cns.14694 (PMC10966135; doi:10.1111/cns.14694)
Supplement: Supplementary file 1 — Appendix S1 [file CNS-30-e14694-s001.docx]

**Supplementary figure legends and tables**

**Figure S1.** **There was no significant difference in the baseline haematoma volume between the ICH group and the ICH+NBO group. ns, not significant.**

**Figure S2. A mouse model of ICH was successfully established.** (A) Collagenase Ⅶ was used to construct the ICH mouse model. (B) MRI was used to confirm the haematoma in the injected region. (C) Obvious haematoma was visible in brain tissue sections from ICH model mice.

**Figure S3. Experimental design.** (A) Schematic illustration of the experimental design, including NBO treatment, ICH induction, behavioural tests and tissue collection.

**Figure S4. Gene score feature plots showed that microglia-mediates synaptic pruning after ICH.** Cells are coloured according to the score for the synaptic pruning signature (representing upregulated DEGs in response to synaptic pruning).

**Table S1. Demographic data and vascular risk factors for intracerebral haemorrhage.**

**Table S2. Multivariate logistic regression analysis of risk factors associated with poor clinical outcomes after intracerebral haemorrhage.**

**Figure S1**


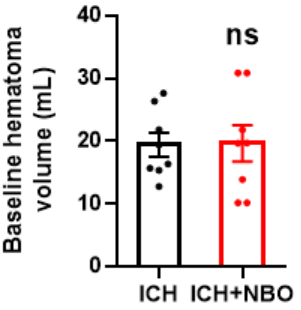


**Figure S2**


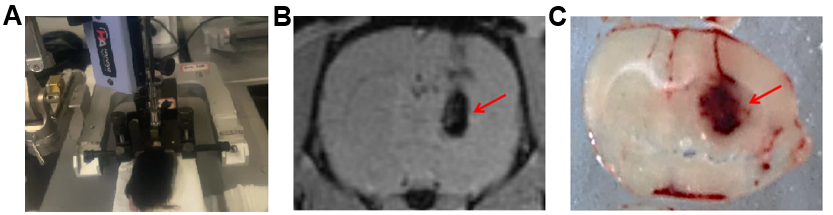


**Figure S3**


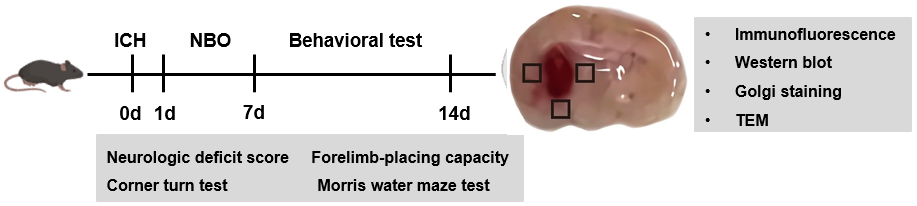


**Figure S4**

**
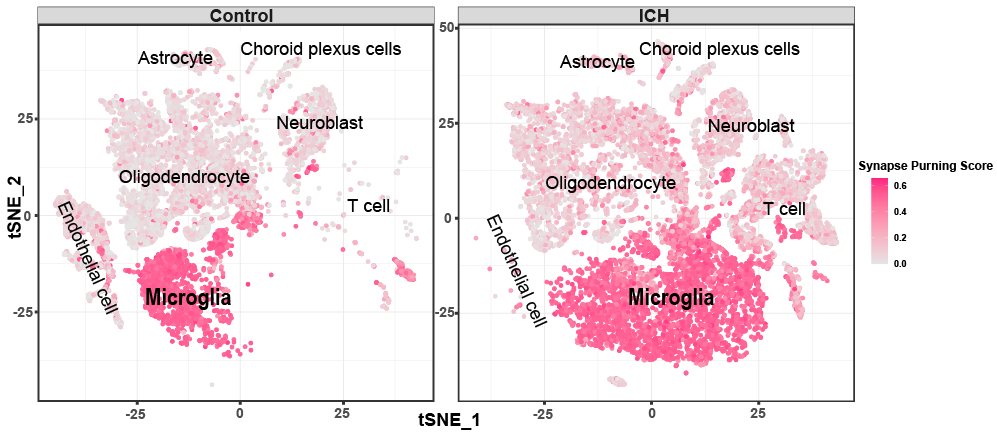
**

**Table S1.** **Demographic data and vascular risk factors of the intracerebral hemorrhage (ICH) patients and control patients in this study.**

| **Characteristic** | **Control (n=78)** | **ICH (n=83)** | ***p* value** |
| --- | --- | --- | --- |
| Male, n (%) | 51 (65.38%) | 56 (67.47%) | 0.779 |
| Age, years (mean ± SD) | 64.13 ± 11.86 | 63.73 ± 13.52 | 0.834 |
| Hypertension, n (%) | 0 | 67 (80.72%) | < 0.001 |
| Diabetes mellitus, n (%) | 0 | 12 (14.46%) | < 0.001 |
| Current smoking, n (%) | 19 (24.36%) | 18 (21.69%) | 0.687 |
| Alcohol consumption, n (%) | 14 (17.95%) | 13 (15.66%) | 0.698 |
| Plasma glucose level (mmol/L) | 5.44 ± 0.92 | 9.21 ± 2.94 | < 0.001 |
| Plasma potassium level (mmol/L) | 4.00 ± 0.30 | 3.79 ± 0.54 | < 0.01 |
| Blood leucocyte count (×10^9^/L) | 5.93 ± 1.38 | 9.64 ± 4.47 | < 0.001 |

**Table S2. Multivariate logistic regression analysis for risk factors of poor clinical outcomes after intracerebral hemorrhage.**

| **Logistic analysis**  **(*p-*value)** | **Hematoma**  **volume** | **NIHSS**  **score** | **GCS**  **score** |
| --- | --- | --- | --- |
| Age | 0.231 | 0.179 | 0.536 |
| Gender | 0.207 | 0.255 | 0.032* |
| Hypertension | 0.140 | 0.788 | 0.428 |
| Diabetes mellitus | 0.685 | 0.645 | 0.726 |
| Current smoking | 0.675 | 0.527 | 0.967 |
| Alcohol consumption | 0.246 | 0.224 | 0.138 |
| Plasma glucose level | 0.518 | 0.384 | 0.548 |
| Plasma potassium level | 0.276 | 0.578 | 0.895 |
| Blood leucocyte count | 0.157 | 0.005* | 0. 040* |
| Plasma C3 level | **0.006*** | **<0.0001*** | **0.0002*** |

Results were showed as *p* value according to the binary logistic regression analysis and *p* < 0.05 was indicated as asterisk (*).
